# Prevention and treatment of catheter-related venous thrombosis in long-term parenteral nutrition: A SINuC position statement

**DOI:** 10.3389/fnut.2023.1106327

**Published:** 2023-02-06

**Authors:** Vincenzo Zaccone, Luca Santoro, Emanuele Guerrieri, Ilaria Diblasi, Ilaria Roncarati, Giovanna Viticchi, Pietro Vecchiarelli, Angelo Santoliquido, Francesca Fiore, Alessio Molfino, Francesco Landi, Gianluca Moroncini, Antonio Gasbarrini, Maurizio Muscaritoli, Lorenzo Falsetti

**Affiliations:** ^1^Internal and Emergency Medicine, Marche University Hospital, Ancona, Italy; ^2^Department of Cardiovascular and Thoracic Sciences, Fondazione Policlinico Universitario Agostino Gemelli IRCCS, Rome, Italy; ^3^Emergency Medicine Residency Program, Marche Polytechnic University, Ancona, Italy; ^4^Neurological Clinic, Marche Polytechnic University, Ancona, Italy; ^5^Intensive Care Unit, Ospedale di Belcolle, Viterbo, Italy; ^6^Università Cattolica del Sacro Cuore, Rome, Italy; ^7^Department of Geriatrics and Orthopedics, Fondazione Policlinico Universitario Agostino Gemelli IRCCS, Rome, Italy; ^8^Department of Translational and Precision Medicine, Sapienza University of Rome, Rome, Italy; ^9^Clinica Medica, Marche University Hospital, Ancona, Italy; ^10^Department of Medical and Surgical Sciences, Fondazione Policlinico Universitario Agostino Gemelli IRCCS, Rome, Italy

**Keywords:** nutrition—clinical, catheter—complications, thrombosis, parenteral, treatment

## Abstract

The implementation of long-term parenteral nutrition (PN) often requires the placement of central venous access, a procedure that carries a considerable risk of catheter-related venous thrombosis (CRT). The occurrence of CRT represents a major event in the natural history of patients in PN since it can lead to central venous access loss and PN failure. Despite the importance of this topic in clinical nutrition, the prevention and treatment of CRT in PN represents one of the “gray areas” of the literature of the presence of few randomized controlled clinical trials and the generally low level of evidence of published scientific papers. Through a narrative review of the literature and a Delphi consensus, the Italian Society of Clinical Nutrition and Metabolism (SINuC) aimed to collect some practical recommendations regarding the current state-of-the-art in the prevention, diagnosis, and treatment of CRT in patients undergoing long-term PN.

## 1. Introduction

Catheter-related venous thrombosis (CRT) is a frequent complication in patients undergoing parenteral nutrition (PN), with an estimated incidence of 0.045/catheter/year (1). The optimal strategy for preventing and treating this issue is still uncertain due to the absence of high-quality clinical trials and the extreme heterogeneity of performed studies. However, it is necessary to optimize the treatment of this complication since the CRT is a severe event of the natural history of PN (2).

The Italian Society of Clinical Nutrition and Metabolism (SINuC) developed—through a systematic review of the literature and a Delphi assessment of the selected indications—clinical recommendations on preventing, diagnosing, and treating CRT in PN patients. Given the small number of performed trials in this setting and the low level of scientific evidence available on this topic, the panel’s recommendations will be based not only on CRT but also extrapolated from studies performed on deep venous thrombosis of the lower extremities (LEDVT) and of the upper extremities (UEDVT), not specifically related to CRT.

## 2. Materials and methods

The study group used a RAND Delphi method, developed and reported according to existing guidelines (3). Problem areas were identified by the steering group (V.Z.; F.L.; G.M.; A.G.; M.M.) that identified the following topics of interest in catheter-related thrombosis: (i) definition (ii) risk factors, (iii) clinical manifestations and complications (iv) clinical and instrumental diagnosis, (v) treatment, (vi) prevention. For each area, the steering group prepared specific clinical questions to be answered by the current paper. The review group (V.Z., L.F., L.S., A.G., A.S., F.F., I.D., I.R., E.G., G.V., P.V. and A.M.) identified the MeSH major terms for the pre-specified topics and performed the literature search in PubMed/Medline and Web of Science for guidelines, randomized controlled trials, meta-analyses, reviews, case series and original research articles from 1 January 1991 to 1 December 2021. The review group assessed MeSH major terms and considered: “Thrombosis” [MeSH], “Anticoagulation” [MeSH], “Prophylaxis” [MeSH], “Deep vein thrombosis” [MeSH], “Catheter related thrombosis” [MeSH], “upper extremity thrombosis” [MeSH], “lower extremity thrombosis” [MeSH], “fibrinolysis” [MeSH], “low molecular weight heparin” [MeSH], “warfarin” [MeSH], “direct oral anticoagulants” [MeSH], alone or in combination. The review group included articles from the last 10 years to obtain more up-to-date information, not excluding older, highly referenced reports. At the end of the extensive literature research, the writing committee selected the list of articles and prepared the recommendations for each domain of interest according to the selected literature. The indications and the supporting studies were submitted to the expert panel for the Delphi rounds. To warrant internal validity, 10 panelists were involved in a two-rounds Delphi assessment through a web platform. In the first Delphi round, for each indication the panel rated the agreement level with a 3-point Likert scale, (1 = highly inappropriate, 2 = undecided, 3 = highly appropriate). Data were analyzed with Kendall’s W concordance coefficient: a recommendation was considered as approved with an agreement for W > 0.7. Items with W lower than 0.7 were submitted again at the second round. The second Delphi round was adopted only to reassess the consensus for the items requiring clarification or without agreement. Only indications reaching a significant agreement after the first or both rounds were included in the final document.

### 2.1. Description of the panel

The panel was composed by Italian specialists involved in the placement and management of central vein catheters and enteral and parenteral nutrition. It was composed by (a) Internal Medicine specialists with a particular experience in angiology, especially in the management of catheter-associated thrombosis (V.Z.; L.S.; A.S.; F.F.; L.F.); (b) Emergency Medicine specialists with a particular experience in venous catheter positioning and management (P.V.; L.F.); (c) Nutrition specialists with a particular experience in enteral and parenteral nutrition and management of long-term catheters (A.M.; M.M.); (d) Internal Medicine experts with a broad experience in hemostasis, thrombosis and nutrition (F.L.; G.M.; A.G.; M.M.).

## 3. Results

### 3.1. Phase I: Evidence review

The review panel selected 75 papers of interest: the largest part (35 papers, 46.7%) was represented by observational (retrospective or prospective) non-interventional studies, 22 (29.3%) papers were reviews (systematic or narrative) 7 (9.33%) works were randomized controlled trials. The review panel included also 10 (13.3%) clinical guidelines. From the analysis of the selected papers, the panel prepared 71 clinical recommendations.

### 3.2. Phase II: Delphi rounds

Seventy-one indications were selected by the review panel and submitted to the Delphi process. At the end of the first round, 53 reached a significant agreement, 6 were asked for a second round, while 13 were discarded. In the second round, the panelists reached an agreement for 5 recommendations, while one item did not reach the consensus and was discarded.

## 4. Definitions and epidemiology

• *Definition* (Table 1): UEDVT is defined as a deep vein thrombosis (DVT) involving brachial, axillary, subclavian, and anonymous veins (1). This definition excludes the veins of the superficial venous circulation (cephalic vein, antecubital vein, basilic vein), for which we will adopt the term superficial thrombophlebitis. LEDVT involves the deep venous circulation of the lower limbs (popliteal and sub-popliteal veins, common, superficial, and deep femoral veins, iliac veins) (1). This definition excludes the superficial venous circulation (saphenous and collateral veins) for which superficial thrombophlebitis is more appropriately defined. CRT refers to superficial or deep venous thrombosis related to the presence of a peripheral or central venous catheter. Some of these venous thromboses may involve superficial veins, as in the case of CRT related to peripheral vein catheters. Other thromboses may involve deep veins, as in the case of CRT related to midline catheters, peripherally-inserted central catheters (PICC), centrally-inserted central catheters (CICC), port, PICC-port, and femorally-inserted central catheters (FICC); in the latter case, we speak of CRT, which are those of greater clinical relevance. Among the CRT, we can distinguish (a) DVT of the upper limb related to the use of midline, PICC, PICC-port, CICC, thoracic port (CR-UEVDT), and (b) deep vein thrombosis of the lower limb related to catheters inserted in the femoral veins, as FICC and FICC-port (CR-LEVDT). Given the infrequent clinical use of femoral catheters, CR-LEVDT is far less frequent than CR-UEVDT, and we will refer more generally to CRT, including all deep vein thrombosis related to venous catheters.
• *Epidemiology* (Table 2): Incidence data regarding CR-UEDVT are uncertain, as they vary according to the study design and enrolment criteria, being on average 1 case/100,000/year (3–4% of the total DVT) (4–6). In any case, it is worth underlining that CR-UEDVT make up about 85% of the total number of UEDVT (4), and the progressive increase in the incidence of UEDVT over the last 30 years (7, 8) is due to the increasing use of venous catheters. CRT incidence in patients undergoing PN is derived from large, retrospective cohort studies and is estimated at 0.02–0.09 cases/catheter/year (9) or 0.12/1,000 catheters for every day (10, 11). A recent prospective study (1) is in line with previous literature data. This datum, however, if compared to the number of catheters placed, is significantly reducing over the last 20 years because of improved techniques for placement and improving management of central venous catheters (11). FICC is associated with high risk of infection and CR-LEDVT (12).

**TABLE 1:** What are the definitions in the setting of catheter-related thromboses?

*N*	Recommendation
1.1	UEDVT should be defined as a DVT involving brachial, axillary, subclavian, and anonymous veins
1.2	UEDVT definition should not include cephalic vein, antecubital vein and basilic vein
1.3	LEDVT should be defined as a DVT involving popliteal and sub-popliteal veins, common, superficial, and deep femoral veins, iliac veins
1.4	LEDVT definition should not include saphenous and collateral veins
1.5	CRT definition should involve deep veins of upper and lower limbs related to catheter placement

CRT, catheter-related thrombosis; DVT, deep vein thrombosis; UEDVT, upper extremities deep vein thrombosis; LEDVT, lower extremities deep vein thrombosis.

**TABLE 2:** Which is the prevalence of CRT according to catheter site?

*N*	Recommendation
2.1	Catheter-related UEDVT incidence is uncertain but is a rare event, representing 3–4% of the total DVT
2.2	Catheter-related UEDVT represents 85% of all the UEDVT
2.3	FICC should be deemed to be associated with a higher risk of infection and catheter-related LEDVT

CRT, catheter-related thrombosis; FICC, femorally inserted central catheter; UEDVT, upper extremities deep vein thrombosis; LEDVT, lower extremities deep vein thrombosis.

## 5. Pathogenesis and risk factors

The pathogenesis of thrombosis is multifactorial (11). The traditionally recognized pathogenic factors are endothelial damage, reduced venous blood flow, and a hypercoagulable state, all considered in Virchow’s classic triad (13). While the first two factors should be considered partly technical and, therefore, susceptible to correction, a hypercoagulable state that depends on the intrinsic characteristics of the patient and his comorbidities (i.e., oncological diseases, thrombophilic states, sepsis, pregnancy or peripartum, and reduced mobility), is little modifiable. CRT should not be confused with fibroblastic sheathing (FS), either in therapeutic or clinical terms: while thrombosis is a tissue originating from endothelial damage and, as such, morphologically correlated with the vessel wall, the sheath is a phenomenon originating from the intravascular foreign body and therefore, morphologically correlated with the catheter. FS formation begins early after catheter insertion and is completed in 2 weeks (14). According to the currently most accepted theory (15), within 24 h of insertion, fibronectin, a circulating protein produced by the liver, begins to deposit on the surface of the catheter, which in turn attracts macrophages; these adhere to the catheter, differentiate into smooth muscle cells and fibroblasts and begin to produce collagen, thus creating a connective tissue that slowly envelops the catheter. Under this light, FS can be interpreted as a foreign body reaction of blood tissue to the catheter (14). On the other hand, thrombosis begins with tissue factors released after endothelial damage; in fact, the thrombus is formed by platelets aggregates and activated coagulation proteins, including fibrin, aimed at repairing vessel damage. Although the pathogenetic mechanisms of FS and thrombosis formation are therefore different, their relationship is still unclear (16). In several clinical studies (17) the two phenomena appear distinct and unrelated. Both CRT and FS are pathophysiological phenomena that constantly occur after placing any venous catheter, although, in most cases, they remain subclinical and below the instrumental diagnostic possibilities used in clinical practice. The course is different when they become symptomatic. Whereas a symptomatic CRT presents with the clinical picture of venous thrombosis and the potential risk of pulmonary embolism and other severe clinical outcomes, an FS may be associated with at its worst catheter malfunction. The mechanisms by which subclinical CRT can become clinically manifest are not clear. In fact, the different responses to endothelial damage likely depend on a combination of co-factors such as venous stasis and hypercoagulability (Table 3). For example, the placement of a catheter of significant caliber in the vessel (occupying more than 33% of the diameter of the vein) (18) and the infusion of fluids is associated to a critical blood flow slowdown, encouraging thrombus formation. Moreover, factors related to the patient’s underlying pathology, such as active cancer, inflammatory states, and thrombophilia, which can further promote the thrombogenic process. However, in this setting, the risk factors associated with the catheter choice, implantation, and management techniques are interesting and unique since they are more useful in preventive strategies (19). In particular, the correct selection of the catheter with the most appropriate external diameter for the selected vein seems a critical factor to prevent CRT. Catheter diameter plays an essential role in CRT: in fact, the presence of the catheter within the vessel and the infusion of solutions causes blood flow slowing, as in the case of subject with small veins, as children (20, 21). The ideal ratio between the catheter’s outer diameter and the vein’s diameter has not yet been defined. However, there is general agreement that the outer diameter of the catheter should not exceed 33% of the diameter of the vein (19). In practical terms, the most suitable catheter for a 3 mm vein is 1 mm (3 Fr), for a 4 mm vein, it is 1.3 mm (4 Fr), and so on (22). Current guidelines (23) suggest that up to 45% occupation of the internal vessel diameter is possible, but a rule such as the one suggested above (Fr of the catheter should be equal to or less than mm of the vein) is safer and easier to adopt. Catheter material may also represent a risk factor. Polyethylene and polytetrafluoroethylene catheters have been shown to be more thrombogenic than silicone or polyurethane catheters (22); it is important to emphasize that these materials are now the only ones used for peripheral catheters. Central catheters—more commonly used for PN—are made of silicone or polyurethane, two materials not differing in terms of thrombotic risk. There is no convincing evidence of the real effectiveness of materials with an antithrombotic effect (24, 25). Another factor that reduces endothelial trauma is the use of micro-introduction kits with thin (21G) needles and small diameter (0.018′′) mini-guides made of nitilon (nickel-titanium), with a soft, straight tip, otherwise known as a floppy straight tip (26). The latter, particularly thin and atraumatic, minimizes the risk of endothelial damage, which is common with 0.035′′ metal guides, especially with j-tips. Another risk factor for CRT related to central venous catheters is inadequate central tip position (2). Catheters with a proximal tip positioned within the superior vena cava or even in an anonymous vein are associated with an increased risk of CRT. Indeed, guidelines now recommend intra-procedural methods of tip placement (such as intracavitary ECG, cardiac ultrasound assessment, or, in special cases, fluoroscopy) to improve the accuracy of the central position. A short catheter, with the tip positioned against the cavity wall or the wall of an anonymous vein, will cause local endothelial injury by a dual mechanism, mechanical (trauma against the vessel wall) and chemical (infusion of irritants—such as hyperosmolar parenteral nutrition—directly against the endothelium). A correct catheter fixation is another item that can help reduce phlebitis, local infections, and catheter migration, all risk factors for developing CRT (27). Particularly, inadequate fixation is the cause of possible catheter dislocation and catheter micro-movements (“in and out”) in the emergency site, which, as well as encouraging bacterial contamination by the extraluminal route (28), are also associated with continued endothelial trauma. Appropriate choice of the emergence site (with a preference for the middle third of the arm and the infra-clavicular fossa), the use of sutureless skin adhesion devices or subcutaneous anchoring devices (Subcutaneously Anchored Systems, or SAS), and the use of semi-permeable transparent dressings represent the best strategies for correct catheter fixation. A recent meta-analysis showed that using the latter may also reduce Catheter-Related Bloodstream Infection (CRBSI) (29). The pathogenesis of CRT is linked to endothelial damage, which occurs in two ways: either from the venipuncture site (as is the case in most cases) or near the catheter tip (if this is mispositioned). Regarding the first mechanism, since endothelial trauma cannot be avoided when placing a venous catheter, the transition from subclinical CRT to clinically diagnosable or even symptomatic CRT will depend on the extent of the vessel trauma, the presence of local predisposing factors (as venous stasis) and the presence of predisposing systemic factors (as thrombophilic states). Regarding the second mechanism, simple mispositioning will be necessary and sufficient reason for developing clinically manifest CRT, especially for catheters used for PN.

**TABLE 3:** Which are the risk factors to develop CRT?

*N*	Recommendation
3.1	A catheter larger than 33% of the diameter should be considered a risk factor for CRT
3.2	Active cancer, inflammatory states and thrombophilia should be considered as factors that further promote CRT
3.3	Chemical and mechanical endothelial damage should be considered as a risk factor for CRT
3.4	Local inflammation should be considered as a risk factor for CRT
3.5	There is no convincing evidence to suggest antithrombotic material over classical materials to prevent CRT
3.6	Micro-introduction kits should be considered to reduce CRT risk
3.7	Intraprocedural methods of tip placement (such as intracavitary ECG, cardiac ultrasound assessment, or, in special cases, fluoroscopy) should be considered to improve the accuracy of the central position and reduce CRT risk
3.8	A correct catheter fixation should be considered as useful to reduce CRT risk

CRT, catheter-related thrombosis; ECG, electrocardiogram.

## 6. Clinical manifestations and complications of CRT

• *Clinical manifestations* (Table 4): The clinical manifestations of CRT may vary. Literature data underline that more than half of patients are asymptomatic (30). However, the proportion of an asymptomatic state in PN subjects seems to be lower (1). In symptomatic cases, the most frequent signs and symptoms are the classic DVT signs, such as edema, erythema, pain, discomfort, and weakness of the affected limb; the presence of superficial venous circles (Urschel’s sign) is uncommon [CIT]. If the thrombosis involves the anonymous vein or the superior vena cava, symptoms will also affect the neck and face with associated headache and drowsiness until the development—in severe cases—of superior vena cava syndrome [CIT]. In a small proportion of cases, CRT may present as a venous catheter malfunction, occurring when the catheter tip is mispositioned (primary or secondary misposition). It is necessary considering that the isolated presence of this phenomenon, in the absence of clinical manifestations, depends in most cases on phenomena that have nothing to do with CRT: occlusion of the catheter by clots, drugs, lipids or fibroblast sheath, or extraluminal obstruction (as, for example, pinch-off syndrome, kinking or folding of the tip on the vessel wall). A correct diagnosis of the cause of catheter dysfunction requires instrumental examinations, which sometimes can be invasive (echocardiography, computed tomography scan, catheter-gamma). If the catheter cannot be withdrawn but maintains the ability to infuse more frequently, the pathogenesis is due to a fibroblastic sheath or a pinch-off syndrome or it is due to a valved catheter with a malfunctioning valve.
• *Short-term complications* (Table 5): Generally, the frequency of UEDVT complications is lower than in LEDVTs, and it is further reduced when considering CRT. Possible short-term complications include pulmonary embolism (PE); extension of thrombosis proximally or distally with phlegmasia cerulea dolens, compartment syndrome, superior vena cava syndrome; catheter infection, and sepsis.
• *Pulmonary embolism:* in general terms, PE risk in patients with UEDVT is lower, but still significant, than in LEDVT when analyzing catheter-related events. Registry analyses performed in unselected cohorts of subjects with UEDVT have demonstrated a lower incidence of clinically significant PE than in LEDVT (3% vs. 16%, 9% vs. 29%, 14% vs. 51%) (5, 31, 32). A further study confirmed an overall low incidence of PE among both UEDVT (5%) and CRT (1%) patients (4). The incidence of PE is even lower for PICC-related UEDVTs than for CICC-related DVT (33). However, the risk of embolization relates to the site of thrombosis and the patient’s general clinical condition, being more significant in patients with severe thrombophilic conditions such as cancer or congenital and acquired thrombophilia (34).
• *Other short-term complications: Phlegmasia cerulea dolens* and compartment syndrome are rare but potentially fatal complications that have been reported among UEDVT (35) and are generally caused by a delay in the diagnosis of thrombosis. Their incidence among catheter-related DVT is rare, if not exceptional. In contrast, superior vena cava syndrome has been reported with a significant incidence in patients in PN due to catheter-related DVT (36). CRT can be complicated by catheter-related infection. However, the association between infection and catheter thrombosis is still debated (37–40). Although there is a theoretical basis to justify this association, no clinical studies have demonstrated a higher incidence of infection in CRT catheters or CRT in catheter-related bacteremia.
• Long-term complications
• *Post-thrombotic syndrome (PTS)*: In general, patients with UEDVT have a lower PTS incidence than those with LEDVT, and this incidence is further reduced in patients with CRT (41). The estimated incidence of PTS after UEDVT varies widely from 7 to 46%. Different definitions from heterogeneous studies (41, 42) drive this variability; in a prospective study enrolling 53 patients with symptomatic UEDVT and receiving anticoagulation therapy alone, the cumulative incidence of PTS at 2 years was 27.3% (43). A recent meta-analysis demonstrated a 14% prevalence of PTS in patients with secondary UEDVT (44). The post-thrombotic remnant was associated with a fourfold increased risk of developing PTS in UEDVT (31). From a preventive point of view, no studies define whether the use of compression garments can reduce PTS risk in catheter-related DVT, and these are of little use in clinical practice (6) and not recommended by the guidelines (45).
• *DVT or PE recurrence*: This event has been estimated between 2.3 and 1.8% at 3 months in UEDVT (for DVT and PE recurrence, respectively) (32), 3.5% at 1 year (6) and 9.8% at 3 years (5) depending on the studies. The major determinants in the risk of VTE recurrence appear to be the presence of symptoms of VTE at diagnosis, active cancer, and age over 55 years (32). No such data exist regarding catheter-related DVT.
• *Venous obstruction:* Development of vascular stenosis is associated with prior catheter-related DVT and may compromise future vascular access placement (46). This may be particularly important in chronic PN subjects requiring the permanent availability of “long-term” venous access (2). However, the only existing clinical data in the literature refer to venous obstruction secondary to long-term dialysis accesses, not to parenteral nutrition accesses [CIT].

**TABLE 4:** Which are the clinical manifestations of CRT?

*N*	Recommendation
4.1	Half of the CRT cases are asymptomatic
4.2	The most frequent signs and symptoms observed in CRT are edema, erythema, pain, discomfort, and weakness of the affected limb, while the presence of superficial venous circles is uncommon
4.3	If the thrombosis involves the anonymous vein or the superior vena cava, symptoms could also affect the neck and face with associated headache and drowsiness until the development of superior vena cava syndrome.
4.4	CRT can be clinically evident only with venous catheter malfunction that occurs when the catheter tip is mispositioned

CRT, catheter-related thrombosis.

**TABLE 5:** Which are the complications of CRT?

*N*	Recommendation
5.1	According to time of appearance after thrombosis, complications should be divided into short-term and long-term complications
5.2	Short-term complications should include PE, DVT extension, phlegmasia cerulea dolens, compartment syndrome, superior vena cava syndrome, catheter infection, and sepsis.
5.3	Long-term complications should include post-thrombotic syndrome, DVT and/or PE recurrence, and permanent venous obstruction

CRT, catheter-related thrombosis; DVT, deep vein thrombosis; LEDVT, lower extremities deep vein thrombosis; PE, pulmonary embolism; UEDVT, upper extremities deep vein thrombosis.

## 7. Clinical and instrumental diagnosis

Catheter-related DVT is commonly asymptomatic, with a prevalence ranging from 19% [in patients studied venous colorDoppler ultrasound (CDU)] to 41% (in patients with CVC investigated at venography) (37). The reliability of these data is distorted by many factors: phlebography itself represents a thrombogenic risk factor and has many false positives; ultrasound examination—if not accurately performed—leads to a misinterpretation of FS as an asymptomatic thrombosis. However, the benefit of treating these asymptomatic thromboses is quite uncertain; therefore, despite the high prevalence, it is currently recommended not to look for signs of catheter-related DVT in patients with CVC (1) routinely. Regarding symptomatic patients, the diagnostic work-up (Table 6), particularly for catheter-related UEDVT, is still unclear and unexplored and is based on low-level evidence, unlike LEDVT (47). Clinical examination appears to be unspecific (30–64%) (42); the presence of pain and edema, in combination rather than alone, however, has proven to be the most accurate diagnostic symptoms (48). Several scores have been developed to increase the diagnostic accuracy of clinical examination, with an approach similar to the Wells score for LEDVT. The currently suggested system is the “Constans clinical decision score” (CCDS), a four-item score with a three-level clinical probability, which has demonstrated moderately satisfactory diagnostic accuracy in the diagnosis of UEDVT but needs further validation (48). Currently, a specific score for CRT has not been developed. In agreement with LEDVT, in the UEDVT clinical decision-making process, D-dimer is useful to exclude the diagnosis, with a high sensitivity (92–100%) and a high negative predictive value. The specificity of D-dimer is generally low (10–64%) (49, 50) and there are currently no studies demonstrating this observation even in the context of UEDVT. However, there are still no clinical studies documenting D-dimer use in diagnosing CRT, and its predictive value in this clinical context is still object of debate. In clinical-laboratory suspicion, the first-choice diagnostic test is venous CDU of the upper limb with compression, which is non-invasive, easy and accurate, and is widely used for diagnostic purposes in clinical practice (6). A systematic review of the literature regarding patients with clinical suspicion of catheter-related DVT defined sensitivity and specificity of 84 and 94%, respectively, for CDU without compression, 97 and 96% for compression ultrasound (CUS), and 91 and 93% for CDU with CUS. These results were confirmed by a recent large, prospective trial that showed high sensitivity and specificity of whole-limb CDU with CUS in the context of UEDVT (51). The method is based on (1) direct visualization of the thrombus; (2) CUS; (3) absence of flow on colorDoppler examination; (4) abnormal flow pattern with reduced or absent velocity variability during the Valsalva maneuver on pulsed Doppler examination. However, several studies showed that the addition of color Doppler ultrasound to compression ultrasonography does not improve the diagnostic accuracy of the CDU examination (52). The venous CDU has limitations: (1) it is an operator-dependent technique; (2) in case of suspected subclavian vein or innominate vein thrombosis, the diagnosis can be difficult because the presence of the overlying bone barrier prevents CUS and the associated acoustic shadow makes Doppler-wave visualization very difficult. Another weakness of the CDU method in the context of CRT is that it can overestimate CRT presence, because the ultrasonographic differential diagnosis between CRT and FS (14) requires special experience and training. Phlebography, which was considered 20 years ago the gold standard for the diagnosis of thrombosis, is today no longer recognized for CRT diagnosis, since it is an invasive, expensive, potentially harmful, and largely inaccurate method due to the high incidence of false positives. Computed tomography (CT) is a second-level method in the diagnostic pathway of catheter-related DVT. It is useful in adult patients, especially in the cases where the thrombus extends into venous vessels that cannot be investigated by ultrasound (superior vena cava, inferior vena cava) or, more rarely, when CDU is not diagnostic. Interestingly, even the CRT diagnosis adopting CT is operator-dependent, and this method cannot differentiate CRT from FS. This differential diagnosis is even more difficult with CT than with CDU. In some cases, the distinction between CRT and FS at the superior vena cava or right atrium level may require echocardiography. Magnetic resonance imaging (MRI) can be considered to investigate complex, central CRT especially in patients allergic to iodinated contrast media (53), however, its costs and its reduced availability reduce the utility of this diagnostic procedure.

**TABLE 6:** Which are the clinical and instrumental approaches to diagnose CRT?

*N*	Recommendation
6.1	The diagnostic work-up, particularly for catheter-related UEDVT, is still unclear and unexplored
6.2	The Constans and the Wells clinical decision scores could be considered to improve diagnosis in UEDVT and LEDVT, respectively; however, these scores are not validated in CRT and their utility is still object of debate. The panel recommends to cautiously use these scores.
6.3	D-dimer could be considered to exclude the diagnosis for its high sensitivity; however, it is not extensively studied in CRT and its utility in this clinical context is still object of debate. The panel recommends to cautiously interpret d-dimer results in this setting.
6.4	CDU with CUS should be considered as a first-line exam to confirm or exclude CRT.
6.5	Contrast-Enhanced CT or MRI scan could be considered if the site of suspected thrombosis cannot be reached by CDU with CUS.

CRT, catheter-related thrombosis; CDU, colorDoppler ultrasound; CUS, compression ultrasound; LEDVT, lower extremities deep vein thrombosis; UEDVT, upper extremities deep vein thrombosis.

## 8. Treatment

The panel identified as goals of CRT treatment (Table 7) are (i) reduction of symptoms, (ii) catheter rescue and, (iii) prevention of short- and long-term complications such as (a) thrombus extension to more central veins and intracranial sinuses, (b) pulmonary and systemic embolization, (c) vessel remodeling and chronic venous obstruction, and (d) reduction of the risk of recurrences (12). There are currently no prospective randomized controlled trials related to the treatment of UEDVT and CRT. Most studies in this topic have been performed retrospectively in single centers, with most of the developed recommendations arising from studies performed on LEDVT or from small, specific observational studies with a low-grade of evidence.

**TABLE 7:** Which are the treatment goals and the general treatment measures in CRT?

*N*	Recommendation
7.1	CRT treatment goals should aim at (i) symptoms’ reduction, (ii) catheter rescue (iii) prevention of short- and long-term complications
7.2	Management of complications should aim at: (a) prevention of thrombus extension to more central veins and intracranial sinuses, (b) reduction of the risk of pulmonary and systemic embolization, (c) reduction of the risk of vessels remodeling and chronic venous obstruction, and (d) reduction of risk of recurrences.
7.3	Limb positioning in antideclive position, rest, cold packs, systemic or topical therapy with anti-inflammatory drugs can be suggested as general measures for pain relief in case of symptomatic CRT.

CRT, catheter-related thrombosis.

• *General management*

° *General principles*: Simple maneuvers, such as limb positioning in antideclive position, rest, cold packs, systemic or topical therapy with anti-inflammatory drugs such as Diclofenac (22) are suggested to treat symptoms.

° *Catheter management* (Table 8): One of the first decisions required when managing CRT is whether to remove the catheter or not. Studies have shown that, in the presence of adequate anticoagulant therapy, removing the thrombosed central venous catheter does not necessarily result in a better clinical outcome (54). For this reason, the indication for catheter removal is not routinely recommended (2, 45, 55), and should be defined according to the patient’s clinical needs, the catheter status, the blood vessels conditions, and the response to anticoagulant therapy. Removal is recommended if the catheter is no longer needed (56). The presence of catheter infection, dysfunction or a poorly positioned catheter should also suggest preferring catheter removal (2). It should be considered that if the catheter dysfunction is due to thrombosis (presence of CRT around the tip of the catheter—a very rare occurrence and usually secondary to mispositioning of the tip), the catheter should be removed. However, if the catheter is well positioned and the dysfunction is related, for example, to a lumen obstruction by clots or lipids, we can attempt to unblock the catheter. This is important if the patient has reduced venous vessels availability or is in the perspective of long-term preservation of the catheter. The first unblocking attempt should be hydraulic: pharmacological unblocking should be carried out as a second option, depending on the nature of the obstruction, for example, using thrombolytics in the case of obstruction by clots or ethanol in the case of obstruction by lipids. However, it is good to remember that the vast majority of catheter-related DVT is associated neither with catheter malfunction nor with catheter-related infection and that, therefore, the catheter can be left in place and used without contraindications. If the physician chooses to maintain the venous catheter, anticoagulation therapy or, at a minimum, thromboprophylaxis should be initiated and maintained until the catheter is removed (45). If catheter removal is indicated, at least 3–5 days of anticoagulant therapy is recommended before removing the thrombosed catheter because of the risk of accidental thrombus mobilization (57).

**TABLE 8:** How should a thrombosed catheter be managed?

*N*	Recommendation
8.1	In case of CRT, catheter removal should be considered if the catheter is no longer needed.
8.2	In case of CRT, the presence of catheter infection, dysfunction or a poorly positioned catheter should suggest catheter removal.
8.3	In case of CRT with thrombosis around the tip, the catheter should be removed after anticoagulation.
8.4	In case of CRT, if the physician chooses to maintain the catheter, full anticoagulation or at least thromboprophylaxis should be warranted.
8.5	In case of CRT, if the physician chooses to remove the catheter, full anticoagulation or at least thromboprophylaxis should be warranted for at least 5 days before removal.

CRT, catheter-related thrombosis; LEDVT, lower extremities deep vein thrombosis; UEDVT, upper extremities deep vein thrombosis.

• *Antithrombotic treatment*

° *Anticoagulant therapy* (Table 9): Since there are no targeted randomized controlled trials, recommendations for anticoagulation therapy of patients with UEDVT and catheter-related DVT are derived mainly from those existing for patients with LEDVT or from small specific studies (2, 45). However, there are still no approved treatment regimens for these patients. International (2, 45) guidelines recommend full anticoagulation therapy for patients with UEDVT or catheter-related DVT at least 3 months after the thrombotic event if the CVC (2, 45, 54) is removed. If the CVC remains in place and continues to be used (the most frequent occurrence), a minimum thromboprophylaxis should continue after 3 months of full anticoagulation and until the removal of the catheter. GARFIELD-VTE registry data confirm that 92.7% of patients with UEDVT are treated with anticoagulant therapy (6). At present, the evidence on anticoagulation therapy in patients with CRT in PN is low and needs further studies (58).

**TABLE 9:** Which anticoagulant regimen should be preferred in case of CRT?

*N*	Recommendation
9.1	In case of CRT, full anticoagulation therapy for at least 3 months after the thrombotic event should be warranted if the catheter is removed.
9.2	In case of CRT, full anticoagulation or thromboprophylaxis (according to individual thrombotic and bleeding risk) should be continued after 3 months of full anticoagulation if the catheter remains in place and continues to be used until the removal of the catheter.
9.3	Existing data confirm a similar duration of anticoagulant therapy for UEDVT and LEDVT. However, data regarding CRT are missing.

CRT, catheter-related thrombosis; LEDVT, lower extremities deep vein thrombosis; UEDVT, upper extremities deep vein thrombosis.

° *Anticoagulant type and dosage*: Registry data tell us that patients with UEDVT are usually treated with low molecular weight heparins (LMWH), dicumarolic agents or direct oral anticoagulants (DOAC); the use of these drugs is similar to that for patients with LEDVT with a slight prevalence of the use of parenteral therapy likely due to a greater presence of neoplastic patients among those with UEDVT. Approximately one-third of UEDVT patients were treated with DOAC similarly to LEDVT patients (6). The criteria for choosing the type of anticoagulant are similar to those already used for LEDVT; in particular, renal and hepatic function, the possibility of drug interactions, patient compliance, and the need for future interruptions for invasive procedures must be considered. The type of anticoagulant (oral or parenteral) in PN patients should also take into account the intestinal absorption capacity: patients with particular intestinal syndromes (e.g., short bowel syndrome or mechanical obstruction) have a poor absorption capacity of oral anticoagulants (warfarin and DOAC) (59, 60) and will therefore require parenteral therapy. Any modulation of the dosage of anticoagulant therapy should also be evaluated according to the definition of the patient’s bleeding risk. The American College of Chest Physicians Guidelines consider it possible to reduce anticoagulation for isolated brachial vein thrombosis at prophylactic doses or for a duration of less than 3 months (45). Since there are no specific data on anticoagulation therapy in catheter-related DVT, the same schedules are adopted as for non-catheter-related UEDVT or LEDVT.

° *Therapy duration*: Since the risk of VTE recurrence is substantially overlapping in UEDVT and LEDVT (5, 32), the duration of anticoagulation therapy should be similar overall between the two districts and depends substantially on consideration of the causative events (45, 55). Accordingly, in CRT, anticoagulation therapy should be continued for at least 3 months after the thrombotic event and continued beyond if the catheter remains in place (although the dosage can be lowered to a prophylactic level) (45). The possible further continuation even after 3 months from the thrombotic event and after the CVC removal should be evaluated according to the residual thrombotic risk of the patient (55, 61). The registry data highlight that in UEDVT, clinicians’ guidance on the duration of anticoagulation therapy is variable but overall in agreement with current guidelines. The GARFIELD-VTE data confirm a similar duration of therapy between UEDVT and LEDVT (6), and the proportion of patients with UEDVT and LEDVT taking anticoagulants at 3, 6, and 12 months, respectively, was 82.6% vs. 87.45%, 66% vs. 72.6%, and 45.7% vs. 54.6%; other numerically more limited studies show a shorter duration of therapy in UEDVT (5). However, it should be emphasized that these data cannot be automatically extended to CRT.

° *Thrombolytic therapy* (Table 10): Anticoagulation therapy alone is effective in achieving clinical outcomes in DVT. There are currently no randomized trials comparing anticoagulation therapy alone with thrombolytic therapy in the treatment of UEDVT and catheter-related DVT. Some studies have evaluated the use of intravenous administration of various thrombolytic agents (streptokinase, urokinase, or rt-PA) with various methods of administration (intravenous or catheter-directed) (62). These studies suggest that thrombolysis can improve venous patency at the cost of an increased hemorrhagic risk. A recent systematic review of the literature on UEDVT showed a reduction in PTS in patients treated with thrombolysis or mechanical decompression compared to patients treated with anticoagulant therapy alone (44). Evidence indicates that catheter-directed thrombolysis (CDT) reduces lower-limb PTS (63). The indication for thrombolytic therapy should therefore be contemplated only in cases in which the additional clinical benefit of the latter compared to anticoagulant therapy alone outweighs the predominantly hemorrhagic risks of such a procedure; moreover, thrombolytic therapy has additional costs and requires a high level of clinical expertise compared to anticoagulant therapy alone. Currently, the criteria for considering the use of thrombolysis are: (i) severe severity of symptoms (as, for example, phlegmasia cerulea dolens or compartment syndrome), (ii) thrombus involving most of the subclavian vein and axillary vein, (iii) symptoms persisting for less than 72 h. If these conditions are present, the patient must have a low risk of bleeding, a good functional status, and a life expectancy of more than 1 year (45). In PN, thrombolysis could be indicated in patients with a poor venous asset when the aim is to save the venous catheter or reduce the risk of chronic occlusion and secondary venous stenosis (46). If there is an indication for thrombolysis to be performed, it should be desirable to use catheter-directed methods precisely because of the lower dose of thrombolytic administered and the consequent reduction in the associated hemorrhagic risk (45). Systemic thrombolysis has been largely abandoned in favor of local CDT. Patients undergoing thrombolysis require anticoagulation therapy comparable to that of non-thrombolysed patients (45). Basically, thrombolysis has very few indications in the treatment of CRT since it is a therapy that is far from harmless, expensive, of uncertain efficacy, and probably efficient only in case of thrombi of recent onset.

**TABLE 10:** When and how thrombolysis should be considered in CRT?

*N*	Recommendation
10.1	Criteria for considering thrombolysis in CRT are: (i) severe symptoms (i.e., phlegmasia cerulea dolens or compartment syndrome), (ii) thrombus involving most of the subclavian vein and axillary vein, (iii) symptoms persisting for less than 72 h, (iv) a low bleeding risk, (v) a good functional status, and (vi) a life expectancy of more than 1 year.
10.2	Catheter-directed thrombolysis should be preferred over systemic thrombolysis in the setting of CRT.
10.3	Thrombolytic therapy should aim to: (i) restore faster catheter patency and (ii) reduce the risk of post-thrombotic syndrome.
10.4	In parenteral nutrition, thrombolysis should be considered in patients with a poor venous asset when the aim is to save the venous catheter or reduce the risk of chronic occlusion and secondary venous stenosis.
10.5	Patients undergoing thrombolysis require an anticoagulation regimen comparable to the one of non-thrombolysed patients.

CRT, catheter-related thrombosis; LEDVT, lower extremities deep vein thrombosis; UEDVT, upper extremities deep vein thrombosis.

• *Interventional therapy* (Table 11)

**TABLE 11:** When and how mechanical procedures and cava filters should be considered in CRT?

*N*	Recommendation
11.1	Mechanical and surgical procedures should be considered only in those patients in whom symptoms persist despite anticoagulant therapy and pharmacological thrombolysis.
11.2	Mechanical and surgical procedures could be considered to improve venous patency restoration, reduce treatment duration, and reduce the incidence of post-thrombotic syndrome, however, the evidence in CRT is limited.
11.3	Inferior vena cava filters should be considered in patients with catheter related LEDVT only in presence of an absolute contraindication to anticoagulant therapy.
11.4	Superior vena cava filters should be considered in patients with catheter related UEDVT only in presence of an absolute contraindication to anticoagulant therapy, in exceptional cases and in specialized centers.
11.5	Cava filters use should be adopted only to prevent pulmonary embolization, since they do not affect thrombosis progression, post-thrombotic syndrome risk and venous-ischemia syndrome.

CRT, catheter-related thrombosis; LEDVT, lower extremities deep vein thrombosis; UEDVT, upper extremities deep vein thrombosis.

° *Mechanical thrombectomy and surgical procedures*: Percutaneous mechanical thrombectomy (PMT) refers to a series of percutaneous procedures to remove thrombus through aspiration, fragmentation, or maceration; it may occur alone or in combination with pharmacologic thrombolysis (PCDT). Surgical procedures include surgical thrombectomy, venoplasty, and venous bypass. These procedures are generally indicated only in those patients in whom symptoms persist despite anticoagulant therapy and pharmacological thrombolysis (55). Small, retrospective studies have shown positive results of PCDT in restoration of venous patency and duration of treatment (64, 65). One randomized controlled trial excluded the benefit of PCDT therapy on the incidence of PTS in patients with LEDVT in the face of an increased risk of bleeding complications (66). Therefore, there is little evidence about the clinical benefit of these treatments and there is no clinical experience on the application of these procedures in catheter-related DVT.

° *Cava filters*: The general indication for cava filter use is the presence of an absolute contraindication to anticoagulant therapy in the presence of DVT (45). Although there are no official indication for the placement of filters in the superior vena cava, in literature there are some experiences of patients with contraindication to anticoagulant therapy treated with cava filters (67, 68). Given the low level of evidence of studies supporting the use of these devices and the risk of severe peri- and post-procedural complications (cardiac tamponade, aortic perforation, and recurrent pneumothorax) (67), it is advisable to reserve this type of procedure only in exceptional circumstances, after careful discussion of the related risks and benefits and in specialized centers; moreover, the insertion of cava filter prevents pulmonary embolization but does not affect the progression of thrombosis, PTS risk and venous-ischemia syndrome. Therefore, the indication for a cava filter is rare in catheter-related LEDVT and even rarer (if not exceptional) in catheter-related UEDVT.

## 9. Prevention

Evidence on CRT prevention is limited. However, given the evidence on LEDVT and studies on risk factors related to the onset of UEDVT and catheter-related DVT, it is possible to provide some general and pharmacological recommendations for the prophylaxis of catheter-related DVT in PN patients (Table 12).

**TABLE 12:** How can CRT be prevented?

*N*	Recommendation
12.1	When placing a catheter, thoracic ports should be preferred over femoral ports.
12.2	When placing a catheter, the caliber should be the smallest possible and in any case the diameter should be less than 33% of the internal diameter of the selected vein.
12.3	When placing a catheter, polyurethane or silicone catheters and ultrasound-guided venipuncture (preferably using micro-introduction kits) should be preferred.
12.4	When placing a thoracic catheter, the proper placement of the tip at the cava-atrial junction by intra-procedural methods such as intracavitary ECG should be checked.
12.5	After catheter placement, the catheter should be stabilized at the site of emergence as best as possible, using various strategies simultaneously, as tunneling, subcutaneous anchoring, clear adhesive membranes.
12.6	It is recommended to remove the venous catheter when it is no longer used.
12.7	It is recommended appropriate nursing to reduce risk of infection and CRT.
12.8	Performing flushes of saline before and after each catheter use is recommended to prevent lumen occlusion.
12.9	Performing flushes with anticoagulants should only be reserved for dialysis or apheresis catheters, not affecting CRT occurrence.
12.10	Pharmacological prophylaxis is not routinely suggested to prevent CRT.

CRT, catheter-related thrombosis; LEDVT, lower extremities deep vein thrombosis; UEDVT, upper extremities deep vein thrombosis.

• *General measures*: Current guidelines recommend adopting some general preventive measures especially related to the implantation of central venous catheters (2). These include: (a) favoring accesses such as thoracic ports, PICC-ports, CICCs, and PICCs rather than FICCs and FICC- ports (68); (b) placing CVCs of the smallest possible caliber and in any case whose diameter is less than 33% of the internal diameter of the encysted vein; (c) using only polyurethane or silicone catheters use ultrasound-guided venipuncture, preferably using micro-introduction kits; (d) verify proper placement of the CVC tip at the cava-atrial junction by intra-procedural methods such as intracavitary ECG; (e) stabilize the catheter at the site of emergence as best as possible, using various strategies simultaneously (tunneling, anchoring underneath the catheter): tunneling, subcutaneous anchoring, clear adhesive membranes); (f) remove as soon as possible the venous catheter that is no longer useful (g) appropriate and specialized nursing reduce the risk of catheter infection and CRT. Whatever the catheter to be inserted, standardization of the placement method (“insertion bundle”) combined with good experience of a dedicated team reduces significantly the CRT risk (12, 69). A significant reduction in UEDVT incidence from 3 to 1.9% has been reported through an intervention involving fewer multi-lumen PICC and a diameter reduction of triple-lumen PICC from 6 to 5 French (70). Materials such as polyethylene and Teflon, as mentioned above, are associated with an increased risk of thrombosis, but it is also true that all CVC on the market today are made exclusively of other materials, specifically polyurethane or silicone (71). There is no evidence that distal valve catheters, such as the Groshong, effectively prevent short-term complications such as CRT (72): there is also evidence that such distal valve catheters are associated with an increased risk of catheter malfunction. It should be remembered that occlusion of the lumen of the catheter by clots is a phenomenon that has no relation to CRT. It is currently recommended to perform flushes of saline (2, 73) before and after each use of the catheter to prevent lumen occlusion. Catheter medication with anticoagulants (heparin or citrate) should only be reserved for dialysis or apheresis catheters and obviously does not affect the prevention of CRT.
• *Pharmacological prophylaxis*: The use of anticoagulation prophylaxis is currently not routinely recommended in the prevention of CRT in patients with CVC (57), and this recommendation also applies to patients in PN (2). However, the administration of anticoagulant prophylaxis, although not routinely recommended, should be evaluated according to the patient’s individual thrombotic risk defined according to current standards. This point will likely remain controversial until further studies will be conducted (74, 75). Therefore, the indication for long-term anticoagulant prophylaxis should be defined on an individual basis by adequately balancing the extent of thrombotic risk (previous thromboembolic episodes, previous CRT) with the risks related to the administration of anticoagulant prophylaxis (bleeding events, thrombocytopenia, or osteoporosis due to heparin).

## 10. Conclusion

The main limitations of this paper are related to the overall low quality of evidence and to the absence of specific studies for CRT in several domains: thus, grading of the level of evidence was not possible and the indications given do represent only an expert consensus arising from existing literature.

CRT is an uncommon complication in patients undergoing PN, associated to loss of central venous access; CRT management still represents a “gray area” of evidence that advocates further studies. The prevention of CRT is based mainly on general measures, and pharmacological prophylaxis should only be considered in patients with a high thrombotic risk. In the presence of clinical suspicion of CRT, the suggested diagnostic method is CDU with CUS. The main treatment of CRT is anticoagulant therapy, although in selected cases other therapies, such as thrombolytic therapy or interventional therapy, can be considered to improve recanalization rates. Catheter removal is not routinely recommended and should be defined according to the patient’s clinical needs and the response to anticoagulant therapy. Further high-quality studies are needed to clarify the role of specific diagnostic and therapeutic interventions in the diagnosis and management of CRT in PN patients.

## Author contributions

VZ, LF, LS, and AM contributed to conception and design of the study. VZ, LF, LS, AG, AS, FF, ID, IR, EG, GV, PV, and AM performed the literature search and selected the studies for the discussion. GM, MM, AG, PV, and AS supervised the study. VZ and LF wrote the original draft. VZ, GM, ID, IR, EG, GV, AM, FL, MM, LS, AS, AG, and LF reviewed the study and approved its final version. VZ, LS, AS, FF, LF, PV, AM, MM, FL, GM, and AG involved in the Delphi panel. All authors contributed to the article and approved the submitted version.
